# Selection on the Colombian paso horse's gaits has produced kinematic differences partly explained by the *DMRT3* gene

**DOI:** 10.1371/journal.pone.0202584

**Published:** 2018-08-17

**Authors:** Miguel Novoa-Bravo, Kim Jäderkvist Fegraeus, Marie Rhodin, Eric Strand, Luis Fernando García, Gabriella Lindgren

**Affiliations:** 1 Genética Animal de Colombia Ltda. Bogotá, Colombia; 2 Department of Animal Breeding and Genetics, Swedish University of Agricultural Sciences, Uppsala, Uppsala, Sweden; 3 Department of Biology, National University of Colombia, Bogotá, Cundinamarca, Colombia; 4 Department of Anatomy, Physiology and Biochemistry, Swedish University of Agricultural Sciences, Uppsala, Uppsala, Sweden; 5 Department of Companion Animal Clinical Sciences, Norwegian University of Life Sciences, Oslo, Norway; University of Illinois, UNITED STATES

## Abstract

The Colombian paso horse, the most important horse breed in Colombia, performs specific and particular gaits (paso fino, trocha, and Colombian trot), which display different footfall patterns and stride frequencies. The breed has been selected for gait and conformation for more than 50 years and we hypothesize that this selection has led to kinematic differences of the gaits that can be explained by different genetic variants. Hence, the aims of the study were: 1. To identify if there are any differences in the kinematic and genetic variants between the Colombian paso horse’s gaits. 2. To evaluate if and how much the gait differences were explained by the nonsense mutation in the *DMRT3* gene and 3. To evaluate these results for selecting and controlling the horses gait performance. To test our hypotheses, kinematic data, microsatellites and *DMRT3* genotypes for 187 Colombian paso horses were analyzed. The results indicated that there are significant kinematic and *DMRT3* differences between the Colombian paso horse’s gaits, and those parameters can be used partially to select and control the horses gait performance. However, the *DMRT3* gene does not play a major role in controlling the trocha and the Colombian trot gaits. Therefore, modifying genes likely influence these gaits. This study may serve as a foundation for implementing a genetic selection program in the Colombian paso horse and future gene discovery studies for locomotion pattern in horses.

## Introduction

### The Colombian paso horse

The Colombian paso horse breed (CPH), also known as Colombian criollo paso horse, is the most important horse breed in Colombia. This breed is likely derived from a mix of Spanish horses brought by the conquerors to America starting in year 1493. This group of horses included the Spanish Jennet horse, which was known to perform ambling gaits [1]. In the beginning of the 20th century, the CPH population consisted of a mix of horses that performed several different stepping gaits [2]. The Colombian paso horses have been intensively selected for their gaits (paso fino, trocha and trot) since the 1980´s [2]. Currently, the CPH breed is traditionally divided into four groups based on that they have been bred as separate populations during at least 30 years, which are not generally crossed, and also, their gaits: Colombian paso fino (CPF), Colombian trocha (CTR), Colombian trocha and gallop (CTRG) and Colombian trot and gallop (CTG) (www.fedequinas.org). Recently, the CPF group has been declared as a national genetic patrimony in Colombia (Law 1842 of 2017, http://es.presidencia.gov.co/normativa). This was the first CPH group distinguished outside Colombia and in the 20th century it was the group with the largest population size.

The studbook, Federación Colombiana de Asociaciones Equinas—Fedequinas, was created in 1984 and has over 220,000 registered horses (21% CTG, 5% CTRG, 46% CTR, and 27% CPF). Fedequinas is composed of 24 CPH associations around Colombia and it groups several hundreds of breeders in the country. According to Fedequinas, 30–50% of all CPHs in the population are registered in the studbook (Personal communication). Since 1995, all registered horses are parentage tested and each horse is also examined for basic conformation parameters of the breed by educated representatives from the breeding associations. In addition, the examiners also guarantee the origin of the hair samples used for parentage testing. All horses registered in Fedequinas have the possibility to participate in competitions in Colombia. For each competition, the horses are separated by horse group (CPH, CTR, CTRG or CTG), sex and age (three categories: 33–42 months, 42–60 months, and more than 60 months), and evaluated by three judges. This gives subjective qualifications of the conformation and gait traits evaluated (www.fedequinas.org).

### The gaits in the Colombian paso horses

A large number of gaits and gait variations can be observed in horses, including the walk, trot, canter, and gallop [3]. There are three breed specific gaits within the Colombian paso horses: paso fino, trocha, and Colombian trot. These walking and symmetric gaits [4] are performed with at least one limb in stance phase and they are highly collected with a high stride frequency. All the gaits of these horses exhibit a high animation and energy expenditure.

The paso fino gait is performed only by the Colombian Paso Fino (CPF) group. It is a lateral sequence four-beat and laterally coupled gait, which has an isochronal beat pattern, and an independent limb movement. Often, there are three limbs in stance phase at the same time (S1 Video). There are some gaits, in other horse breeds, with the same footfall pattern to paso fino as for example the tölt in Icelandic horses [5], marcha picada in Mangalarga-marchador [6], paso fino in Puerto Rican paso fino horses, and peruvian paso in Peruvian paso horses [7].

The trocha gait is performed only by the Colombian trocha (CTR) and Colombian trocha and gallop (CTRG) groups. It is a lateral sequence four-beat and diagonally coupled gait [7], in which the forelimb hit the ground before the contra lateral hind limb, and it has a non-isochronal beat pattern. Often, there are two limbs in stance phase at the same time (S2 Video). Other gaits with a similar footfall pattern as the trocha are foxtrot in Missouri Foxtrotter [8] and marcha batida in Mangalarga-marchador [6].

The trot gait is performed by the Colombian trot and gallop (CTG) group and can be considered a variant of the regular trot. It consists of an isochronal two-beat and diagonally coupled gait, which is highly collected. There are either two or four limbs in stance phase (this is the main difference from regular trot, which has aerial phase) (S3 Video). Additionally, in competitions, CTRG and CTG horses are judged for gallop, which is a variant of the traditional canter. This asymmetric gait is a highly collected canter and it has a non-isochronal beat pattern, with at least one limb, always, in stance phase (this is the main difference from the regular canter) (S4 Video).

### The *DMRT3* gene

The ability to perform alternative gaits is partly due to genetics. In 2012 a premature stop codon in the doublesex and mab-3-related transcription factor 3 gene (*DMRT3*_Ser301STOP) caused by the Chr23:g.22999655C>A SNP was described to affect locomotion pattern in horses [9]. The *DMRT3* gene is part of the *DMRT* gene family which includes some important developmental regulators in animals. These are mainly, but not exclusively, involved in sex differentiation and/or sex determination [10]. The *DMRT3* gene encodes a transcription factor involved in the coordination of locomotor system in vertebrates [9] and has been associated with gait performance and harness racing performance in several gaited and harness racing horse breeds [11–14]. Furthermore, Promerová et al. [15] analyzed 141 horse breeds for the *DMRT3* nonsense mutation, including the four CPH groups (CPF: 80, CTR: 67, CTRG: 4, and CTG: 35), however the *DMRT3* genotypes were not compared with performance nor kinematic data. The frequency of the mutant “A” allele in the four CPH horse groups was 0.94 (CPF), 0.1 (CTR), 0.25 (CTRG), and 0.14 (CTG), respectively [15].

### Aims of the study

The Colombian paso gaits display different footfall patterns, stride frequency, and they have been selected for more than half a century. Also, to our knowledge, there are no reports on kinematic parameters of Colombian paso horse gaits, i.e. whether those gaits should be considered discrete or continuum gaits, as reported for the Icelandic horse breed [16]. In addition, the genotype frequencies for the nonsense mutation in the *DMRT3* gene differed between the four CPH horse groups in a previous study [15]. Therefore, we hypothesize that the selection on the gaits performed by the Colombian paso horse breed has led to kinematic differences that can be explained, at least partly, by the nonsense mutation in the *DMRT3* gene.

Hence, the aims of the study were: 1. To identify if there are any differences in the kinematic variables and genetic variants between the Colombian paso horse’s gaits. 2. To evaluate if and how much the gait differences were explained by the nonsense mutation in the *DMRT3* gene, and 3. To evaluate these results for selecting and controlling the horses gait performance.

Finally, based on the gait variation in the CPH horses, the breed provides an opportunity to gain new knowledge about the effect of the *DMRT3* “Gait keeper” mutation on the kinematics of gaited horses.

## Materials and methods

### Sampling

A total of 187 CPH (CPF = 52, CTR = 58, CTRG = 34, CTG = 43, in total 99 males and 88 females evenly distributed among the groups), born between 2000–2013 were selected based on their participation in Fedequinas national competitions and their performed gait. A visual examination using slow motion videos was performed to confirm the gait classification of the horses. Kinematic measurements for 172 of the 187 horses were taken at several horse farms in Cundinamarca, Antioquia, Quindío, Risaralda, Caldas, Cauca, and Valle del Cauca departments of Colombia, South America. Information about genealogy, horse groups (CPF, CTR, CTRG, and CTG), gaits, sex, birthdate, results from the DNA parentage tests, and microsatellite data (13 markers: AHT4, AHT5, ASB17, ASB2, ASB23, HMS3, HMS6, HMS7, HTG10, HTG4, LEX3, LEX33, VHL20) were provided by Fedequinas. All the horses in this study were used in a previous study on the CPH breed that analyzed microsatellite genotypes from all registered CPH horses [17]. Also, for most of the animals selected (n = 130), at least five different videos of the gaits performed in the competitions were analyzed to establish whether the horses performed a clear footfall pattern or not. This study was approved by the Ethics Committee for Animal Experiments in Uppsala, Sweden with permit number 5.8.18-15453/2017.

### Kinematic measurements

**Fig 1:**
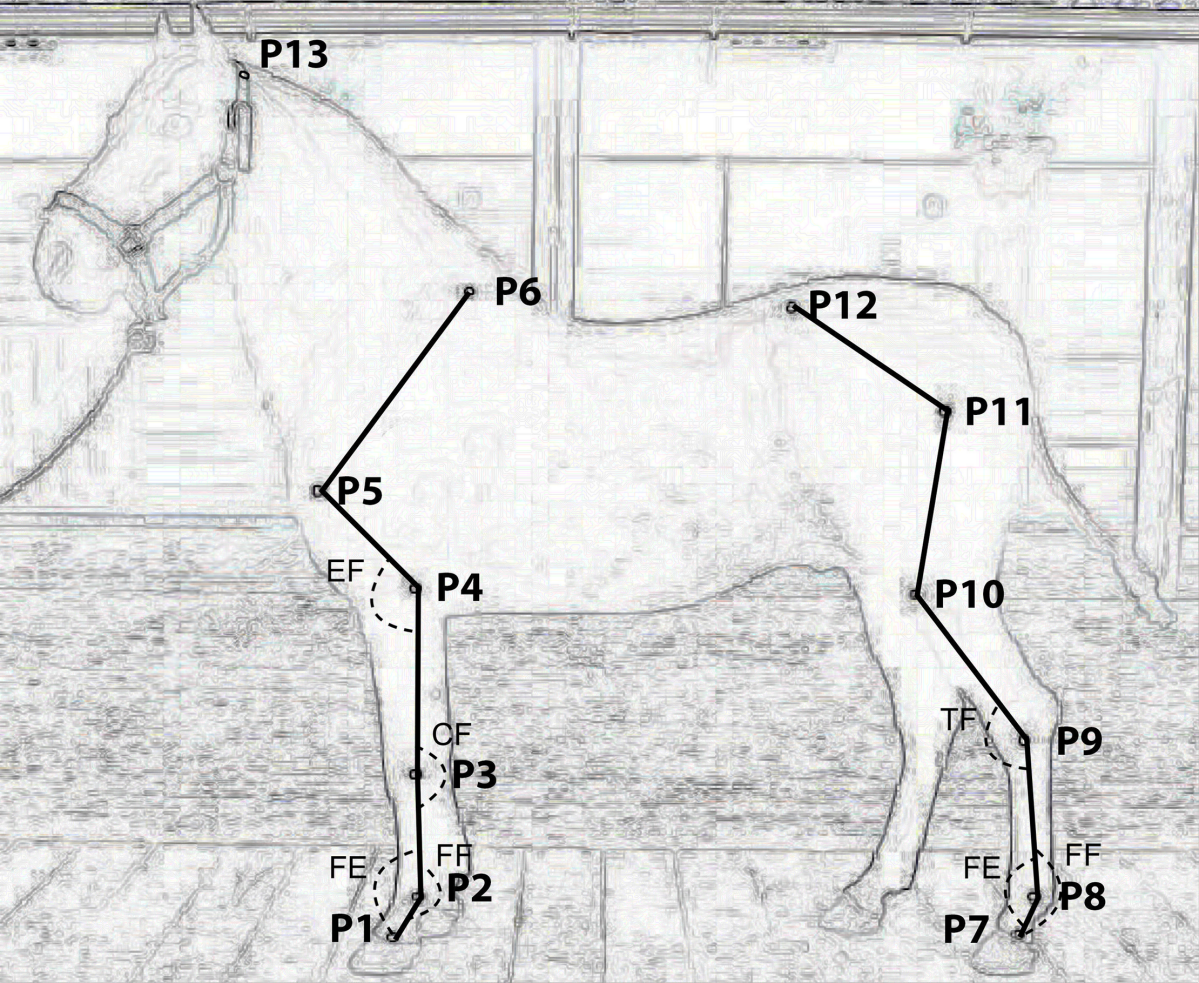
Anatomical landmarks location and angles measured on the horses in motion. P1: Coronary band front, P2: Fetlock front (Metacarpophalangeal joint), P3: Carpal (Carpometacarpal joint), P4: Elbow (Head of radius), P5: Shoulder, P6: Scapula (Top of the withers), P7: Coronary band hind, P8: Fetlock hind (Metatarsophalangeal joint), P9: Tarsus (Tuber calcanei), P10: Stifle (Tibial tuberosity), P11: Hip joint (Summit of trochanter major), P12: Sacro-iliac joint (Tuber coxae), P13: Head (Wings of atlas bone). Angles measured during locomotion, FF—maximum fetlock flexion during the swing phase, FE—maximum fetlock extension during the stance phase, CF—maximum carpal flexion during the swing phase, EF–maximum elbow flexion during the swing phase, and TF–maximum tarsal flexion during the swing phase.

**Fig 2:**
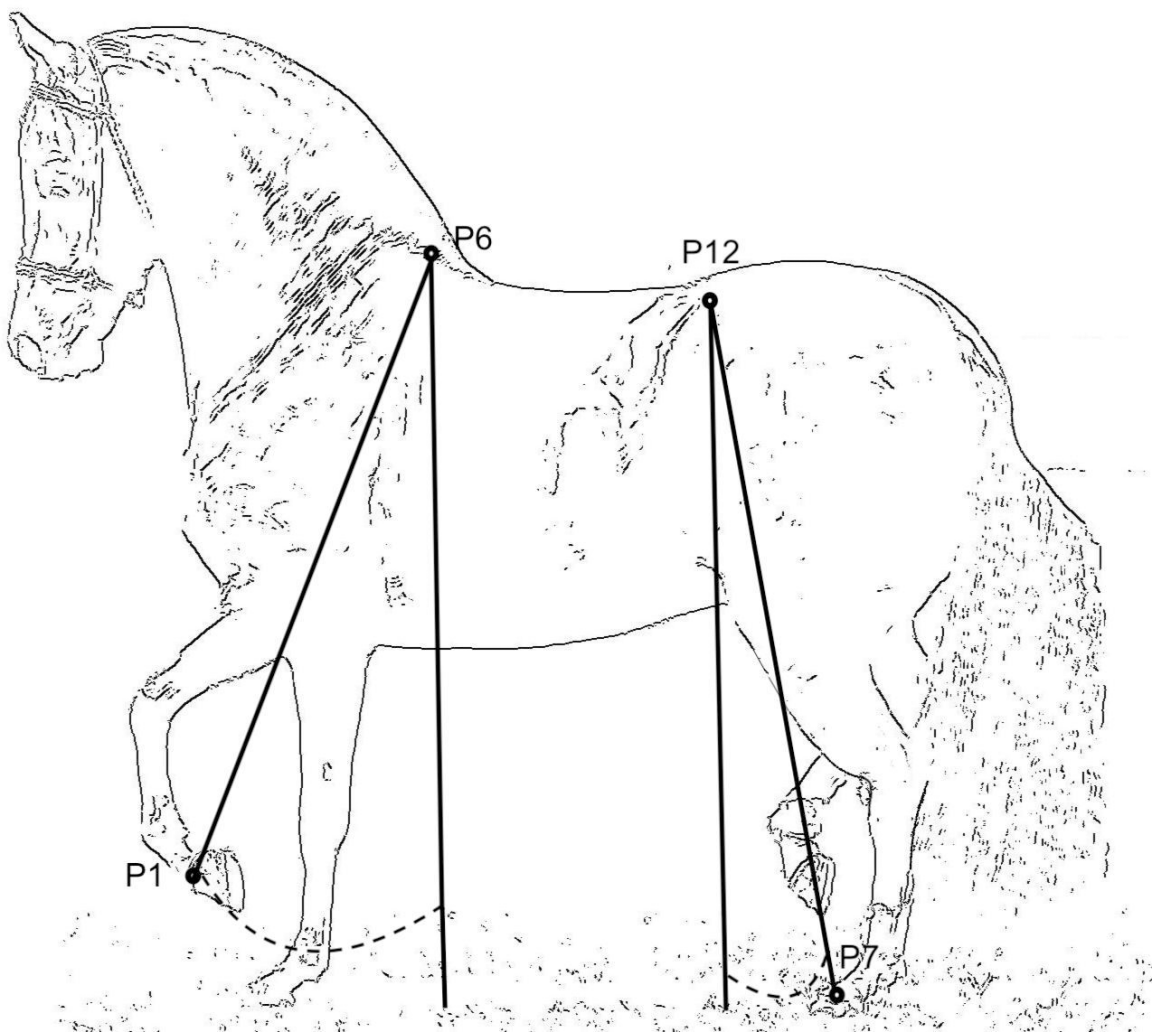
Protraction and retraction limbs angle measurements in a sample of the Colombian paso horse. P1: Coronary band front; P6: Scapula (Top of the withers), P7: Coronary band hind, P12: Sacro-iliac joint (Tuber coxae). The dashed points show the angles measured when the horses were trotting without a rider. Protraction was the maximum angle between P1-P6 and the vertical plane when the forelimb was extended forward. Retraction was the maximum angle between P7-P12 and the vertical plane when the hind limb was extended backwards.

All the kinematic measurements were provided by Fedequinas. Thirteen anatomical landmarks were placed on the horses by the same operator (Fig 1). The landmarks were tracked using the Quintic Biomechanics® software. Measurements were taken for each side of the horse when the horse was in motion. A route was defined for each horse farm and every horse performed (by different riders) its gaits through that route 10 times. Five measurements per side were taken when the horse was perpendicular to the camera. This was recorded by a high-speed camera taking 240 frames per second. A metallic square of 1 X 1 meters was used in all the videos to calibrate the lengths of the measurements. The software automatically calculated the kinematic parameters. These were direct measurements on certain variables in locomotion, such as the angles of the fetlock joint flexion and extension, carpal joint flexion, elbow joint flexion and tarsal joint flexion (Fig 1). Also, the following parameters were measured: stride frequency (strides per minute), stride length (cm), fetlock front speed (cm/s), fetlock hind speed (cm/s) and hock speed (cm/s). The speeds were the mean (per side, left and right) of the maximum speeds registered for each route. The protraction and retraction measurements were defined as explained in Fig 2.

### SNP genotyping

Hair samples from 152 horses of the 187 horses described before (including 15 horses without phenotypic data), were selected from the repository in Fedequinas. To obtain DNA from the hair follicles, a previously reported Chelex–proteinase K protocol was used [11]. SNP genotyping was carried out with the StepOnePlus™ Real-Time PCR System (Life Technologies) using custom designed TaqMan SNP Genotyping Assays (Applied Biosystem) as previously described [9].

### Statistical analysis

#### Selection of the kinematic parameters using asymmetry analysis

**Fig 3:**
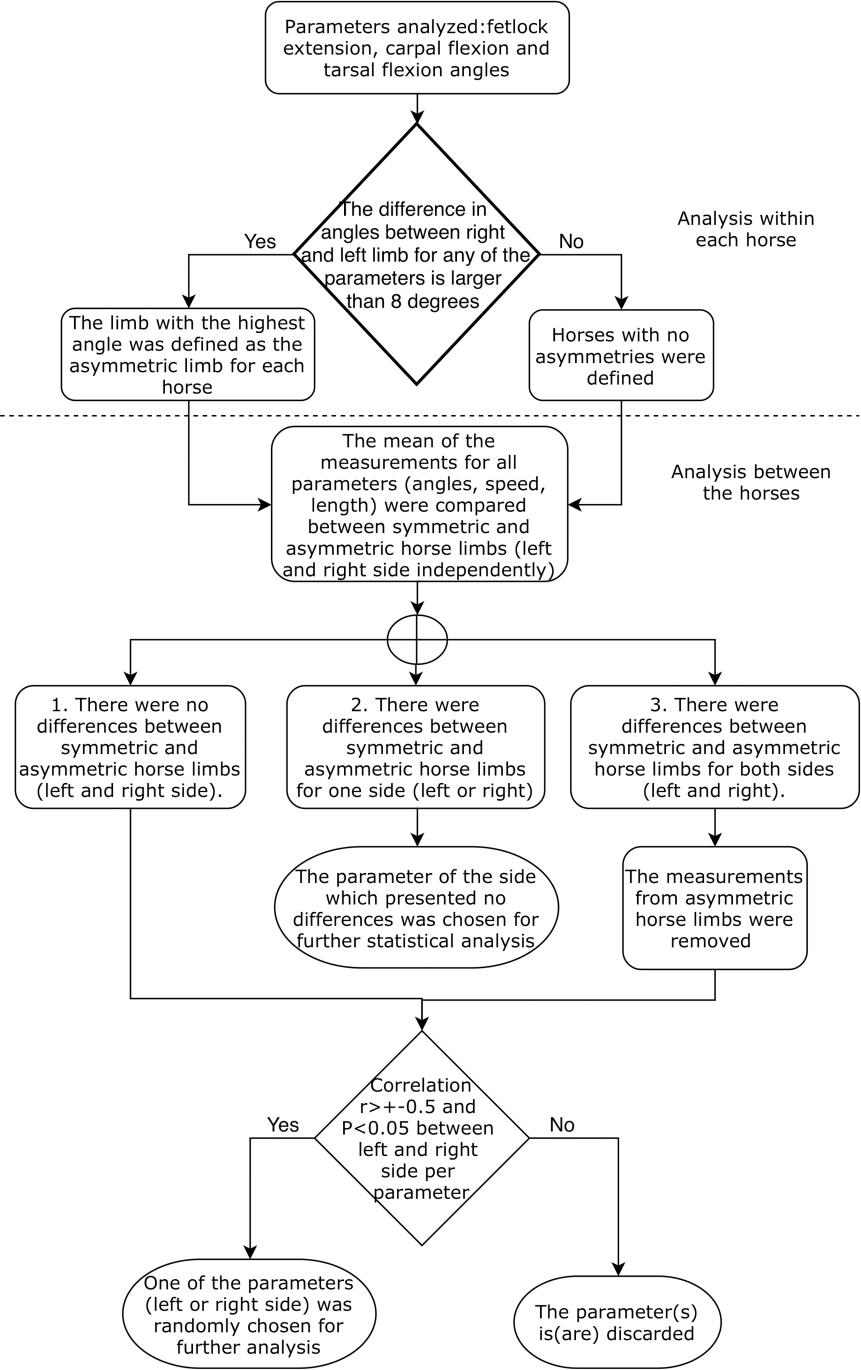
Flowchart of the procedures performed to select one of the kinematic parameters (left or right side of the horse) based on asymmetry analysis for each kinematic parameter in the Colombian paso horses.

The selection of one of the kinematic parameters measured per side (left or right) was based on asymmetry analyses. This preliminary evaluation was done to exclude measurements from lame horses, where asymmetries between kinematic measures from the left and right side can be expected, to avoid possible bias in the further statistical analysis of the kinematic parameters. The flowchart of the process performed is presented in the Fig 3.

The asymmetric limb was identified by comparing the fetlock extension, carpal flexion and tarsal flexion angles between the left and right limb (fore and hind separately) for each horse. The asymmetry per horse and limb was established if the differences between the angles, for any of those parameters, were larger than 8 degrees, and the asymmetric limb was the one with the highest angle (it implies a decrease of the flexion (tarsal and carpal) or the extension (fetlock) of the joints).

Furthermore, the mean of the measurements for all the kinematic parameters (angles, speed or length) between symmetric and asymmetric horse limbs for all the parameters (left and right side independently), were compared by an analysis of variance (ANOVA) using the canova function of the CAR package [18], stratified by horse group and sex. There were three possible scenarios for the ANOVA analysis of all the parameters. 1. There were no differences between symmetric and asymmetric horse limbs (left and right side). 2. There were differences between symmetric and asymmetric horse limbs for one side (left or right). In this scenario, the parameter of the side which presented no differences was chosen for further statistical analyses. 3. There were differences between symmetric and asymmetric horse limbs for both sides (left and right). In this scenario, the asymmetric horse limbs were removed.

For the scenarios 1 and 3 (Fig 3), a Pearson’s correlation coefficient between the left and right side was calculated using the function XI1 of the StatR package [19]. If the correlation was larger than r>+-0.5 and significant (P<0.05), one of the parameters (left or right) was randomly selected for further statistical analyses. If both sides were uncorrelated, that parameter was discarded from further analyses.

#### Mean and variation of the parameters

The statistical analysis was performed in R using the Rwizard software [19]. The mean, confidence interval (CI 95%, using t distribution for size samples < 30), standard error (SE), standard deviation (SD), variation coefficient (VC), skewness and kurtosis were calculated for each variable stratified by horse group and sex, using the StatR package [19]. The normality of all parameters was evaluated with the Shapiro-Wilk test using the function shapiro test of the base stats package.

#### Correlations

The relationships among all variables were estimated with the Pearson’s correlation coefficient using the function XI1 of the StatR package [19]. The strength of the correlations (r) was interpreted based on the guidelines proposed by [20]: 0 to 0.3 (0 to -0.3) = negligible, 0.3 to 0.5 (-0.3 to -0.5) = low, 0.5 to 0.7 (-0.5 to -0.7) = medium, 0.7 to 0.9 (-0.7 to -0.9) = high, 0.9 to 1 (-0.9 to -1) = very high.

#### Analysis of variance

The effects of CPH groups (CPF, CTR, CTRG, and CTG) and sex (females and males) on the parameters, were estimated with analysis of variance (ANOVA) using the canova function of the CAR package [18]. The following model was used for each measurement: *y_ijn_* = *u* + *group_i_* + *sex_j_* + *group* * *sex_ij_* + *e_ijn_*, where *y* is a kinematic measurement for the nth horse, *μ* is the population mean, *group*_*i*_ is the effect of the *i*th horse group (i = 1,…,4), *sex*_*j*_ is the effect of the *j*th sex (j = 1,2), group*sex_*ij*_ is the interaction effect when the *i*th level of group and *j*th level of sex are combined, and *e*_*ijn*_ is a random residual effect. A Kruskal-Wallis test was performed for non-normally distributed parameters using the package dunn.test. The posthoc Tukey contrast test was performed using the function glhtdel of the package multcomp for normally distributed parameters [21]. Dunn’s post hoc test was done after the Kruskal-Wallis test using the package dunn.test. The homoscedasticity of all parameters was evaluated with the Levene test (levene.test function, lawstat package [22]).

#### Multivariate analysis

A discriminant analysis was performed by using a stepwise selection to obtain a subset of the kinematic parameters that best summarized the differences among the groups. This was done with the function candisc of the package candisc [23,24], and ida function of the MASS package [25,26]. The figure of one dimension was obtained with the function plot.cancor of the candisc package [23,24].

#### Genetic analyses

A genetic structure analysis based on 13 autosomal microsatellite markers (AHT4, AHT5, ASB17, ASB2, ASB23, HMS3, HMS6, HMS7, HTG10, HTG4, LEX3, LEX33, VHL20) for 149 of the 187 horses (horses that were genotyped for the *DMRT3* mutation) was performed to evaluate whether the horses were grouped in the same way as in a previous study that analyzed the microsatellite data of the whole registered CPH population [17]. This was done by estimating the number of possible populations in the sample based on Bayesian inference models [27] used by the software STRUCTURE 2.3.4. Also, the analysis of molecular variance (AMOVA) was performed to evaluate the genetic differentiation among CPH groups (CPF, CTR, CTRG, and CTG) with the Arlequin v3.5 software [28].

A Hardy-Weinberg equilibrium (HWE) test for the *DMRT3* genotypes was performed to estimate exact P-values using the Markov chain method with the GENEPOP v.4.3 software [29,30]. The associations between *DMRT3* genotypes and the different CPH groups were evaluated with the Pearson’s chi-squared test using the function VIII1 of the StatR package [19]. The associations between *DMRT3* genotypes and the footfall pattern (whether the horses performed a clear gait or not) in the diagonal gaits (trocha and Colombian trot gaits) were evaluated with Fisher’s exact test using the function VIII2 of the StatR package [19].

## Results

### Statistical analysis

#### Selection of the kinematic parameters using asymmetry analysis

Sixty-nine out of 172 horses with at least one asymmetric limb were found. The kinematic parameters selected for further statistical analysis, based on our method described (Fig 3), are presented in Table 1. The parameters which presented no differences between symmetric and asymmetric limbs (within the left and right side) were highly correlated between both sides (r>+-0.5, P<0.05). Therefore, one side was randomly selected for further statistical analysis (Table 1). Also, for the parameters that presented differences between symmetric and asymmetric limbs for one of the sides (left or right), the parameter that presented no differences between symmetric and asymmetric limbs was selected for further statistical analysis (Table 1). The fetlock extension front in Colombian paso fino-CPF males group was the only parameter that presented differences between symmetric and asymmetric limbs for both sides (left and right). Therefore, the measurements of the asymmetric limbs (n = 5 for the left side and n = 2 for the right side) were removed. After that, both sides were correlated (r>+-0.5, P<0.001), therefore, one of the sides was randomly selected for further statistical analyses (Table 1).

**Table 1:** The kinematic parameters selected based on the asymmetry analysis performed in the CPH breed.

Parameters which presented no differences for both sides in all the groups.	Parameters which presented significant* differences for only one side per group.	Parameters which presented significant* differences for both sides per group.
Carpal flexion	Fetlock extension front in CTG females, and CTR males	Fetlock extension front in CPF males
Elbow flexion	Fetlock extension front in CTRG females and males, and CTR females	
Fetlock flexion—front	Fetlock extension hind in CPF females and CTRG males	
Fetlock flexion—hind	Fetlock front speed in CTRG males	
Hock speed	Fetlock hind speed CTRG males and CTG males	
Protraction	Stride frequency TRGC males	
Retraction	Stride length—front in CTRG females	
Stride length—hind	Tarsal flexion in CPF males and CTRG males	

CPH-Colombian paso horse breed, CPF-Colombian paso fino group, CTR-Colombian trocha group, CTRG-Colombian trocha and gallop group, CTG-Colombian trot and gallop group.

Level of significance

* P<0.05

#### Mean and variation of the kinematics traits

The variation and mean for the kinematic parameters stratified by horse group and sex, are presented in S1 Table. The SD for kinematics parameters was in the range of 0.33–6.07, the largest was found in stride length—hind in CTRG females, and the lowest in protraction—front in CPF males. In general, the variation coefficient for all the parameters was 3.52–33.95. All the parameters, except fetlock front speed (cm/s) and stride length for the front limb (cm) followed a normal distribution.

#### Correlations

Table 2 shows the moderate to high (r>+-0.5) significant (P<0.05) correlations found for the different measurements in the CPH groups. The fetlock flexion for the front and hind limbs as well as the stride length, were the parameters with most correlations with other parameters. The stride frequency had the largest positive correlation with fetlock extension–front (CTRG) and a negative correlation with protraction (CTR).

**Table 2:** The moderate to high significant (P<0.05) correlations between the kinematics parameters measured for each CPH group.

Group	Parameter 1	Parameter 2	r
CTRG	Stride frequency	Fetlock extension—front	0.521
CTR	Stride frequency	Protraction	-0.598
CTR	Elbow flexion	Fetlock extension—hind	0.517
CTR	Elbow flexion	Carpal flexion	0.552
CTRG	Elbow flexion	Carpal flexion	0.522
CTR	Elbow flexion	Retraction	-0.582
CTRG	Fetlock extension—front	Protraction	0.597
CTR	Fetlock extension—front	Retraction	0.594
CTRG	Fetlock extension—front	Retraction	-0.781
CTR	Fetlock extension—hind	Fetlock flexion—hind	0.526
CTRG	Fetlock extension—hind	Tarsal flexion	0.553
CTG	Fetlock extension—hind	Tarsal flexion	0.618
CPF	Fetlock flexion—front	Fetlock flexion—hind	0.545
CTR	Fetlock flexion—front	Fetlock flexion—hind	0.500
CTRG	Fetlock flexion—front	Fetlock flexion—hind	0.633
CTG	Fetlock flexion—front	Tarsal flexion	0.515
CTR	Fetlock flexion—front	Carpal flexion	0.537
CTR	Fetlock flexion—front	Protraction	-0.516
CTRG	Fetlock flexion—hind	Fetlock front speed	-0.502
CTRG	Fetlock flexion—hind	Tarsal flexion	0.535
CTG	Fetlock flexion—hind	Tarsal flexion	0.534
CTR	Fetlock flexion—hind	Protraction	-0.538
CTR	Stride length	Fetlock extension—hind	-0.771
CTRG	Stride length	Fetlock extension—hind	0.535
CTR	Stride length	Fetlock flexion—front	-0.550
CPF	Stride length	Fetlock front speed	0.623
CTR	Stride length	Fetlock hind speed	0.557
CTG	Stride length	Tarsal flexion	-0.513
CPF	Stride length	Hock speed	0.751

Flexions, extensions, protraction, and retraction in degrees, stride frequency in strides per minute, speed in cm/s, stride lengths in cm. CPF-Colombian Paso Fino group, CTR-Colombian Trocha group, CTRG-Colombian Trocha and Gallop group, CTG-Colombian Trot and Gallop group.

#### Analysis of variance

There were significant differences between the CPH groups or sex for most of the kinematic parameters analyzed (Table 3). The fetlock flexion–front, carpal flexion, and strides lengths measurements presented sexual dimorphisms in all groups. Significant interactions effects (P<0.05) between horse groups and sex were found for the stride frequency parameter for all horse groups.

**Table 3:** Differences among the CPH groups or sex on the kinematic parameters analyzed.

Parameter	Horse Group	Sex	Post hoc test^2^
**Kinematics**			
*Fetlock flexion—front*	**		CPF-CTR**, CPF-CTG*
*Fetlock extension—front*	*	**	CPF-CTG*
*Carpal flexion*	***	**	CPF-CTR***, CPF-CTRG***, CPF-CTG***
*Elbow flexion*	***		CPF-CTR***, CPF-CTRG***, CPF-CTG***
*Fetlock flexion—hind*	**		CPF-CTG**
*Fetlock extension—hind*	***		CPF-CTR***, CPF-CTRG***, CPF-CTG***
*Tarsal flexion*	***		CPF-CTG***, CTR-CTG*
*Stride frequency*	***		CPF-CTG***, CTR-CTRG**, CTR-CTG***, CTRG-CTG***
*Fetlock front speed (cm/s)*^1^	***		CPF-CTR***, CPF-CTRG***, CPF-CTG*, CTR-CTRG*, CTRG-CTG***
*Fetlock hind speed (cm/s)*	**		CPF-CTRG**
*Hock speed (cm/s)*	***		CPF-CTR***, CPF-CTRG***, CTRG-CTG*
*Stride length front (cm)*^1^	*	***	CPF-CTR**, CPF-CTRG*
*Stride length hind (cm)*	*	***	CPF-CTR*
*Protraction (degrees)*	**		CPF-CTR**, CPF-CTG*

Flexion and extension in degrees, stride frequency in strides per minute. CPF-Colombian paso fino group, CTR-Colombian trocha group, CTRG-Colombian trocha and gallop group, CTG-Colombian trot and gallop group.

^1^ For a non-normally distributed parameter a Kruskal-Wallis test was done.

^2^ Tukey’s post hoc tests for normally distributed parameter, Dunn’s tests for non-normally distributed parameters after a significant (P<0.05) Kruskal-Wallis test. These tests were done between every pair of horse groups.

Level of significance

* P<0.05

** P<0.01

*** P<0.001.

#### Multivariate analysis

**Fig 4:**
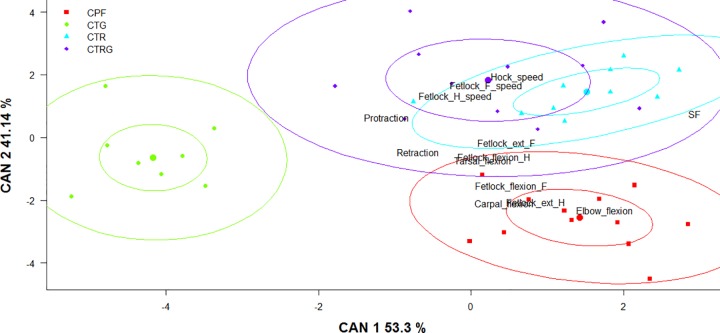
Discriminant analysis for the CPH groups using kinematic parameters. Each point represents an individual classified in one Colombian paso horse group: CPF-Colombian paso fino group; CTR-Colombian trocha group; CTRG-Colombian trocha and gallop group; CTG-Colombian trot and gallop group. F, front; H, hind; ext, extension; SF, stride frequency. Ellipses show 0.5 (inner) and 0.95 (outer) level of significance for each horse group.

**Fig 5:**
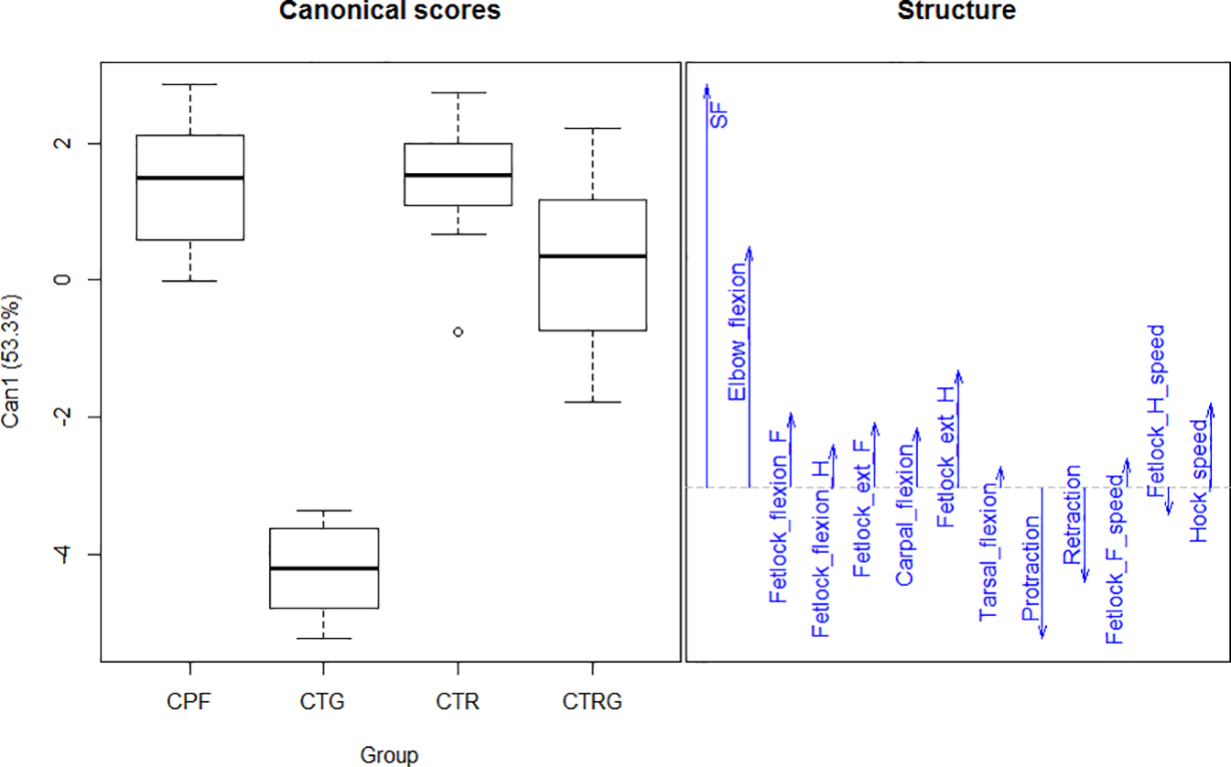
Canonical scores and the parameters that were selected for the first dimension of the discriminant analysis of the Colombian paso horse groups. CPF, Colombian paso fino group; CTR, Colombian trocha group; CTRG, Colombian trocha and gallop group; CTG, Colombian trot and gallop group. F, front; H, hind; ext, extension; SF, stride frequency.

The discriminant analyses resumed 94.44% of the variance in the two first axes based on 13 selected parameters (stride frequency, elbow flexion, fetlock flexion front, fetlock flexion hind, fetlock extension front, fetlock extension hind, carpal flexion, tarsal flexion, protraction, retraction, fetlock front speed, fetlock hind speed, and hock speed) (n = 41, i.e. only the horses that had all the 13 measurements) (Fig 4). The contribution of each parameter to the first dimension is presented in Fig 5. The analysis showed that the Colombian trot is the most differentiated group in the CPH breed. The parameter with the highest contribution to the first discriminant function was stride frequency followed by the elbow flexion. Overall, there were no differences between the CTR and the CTRG groups, except for stride frequency and fetlock front speed, as seen in the ANOVA analysis.

**Fig 6:**
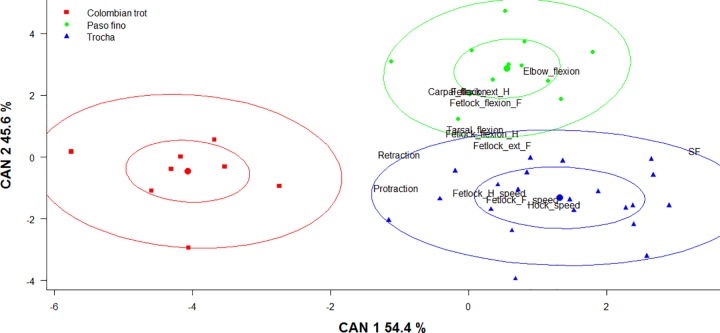
Discriminant analysis between the Colombian paso gaits using kinematic parameters. Each point represents an individual classified per gait. F, front; H, hind; ext, extension; SF, stride frequency. Ellipses show 0.5 (inner) and 0.95 (outer) level of significance for each gait.

In Fig 6 the results of the discriminant analysis are presented per gait instead of horse groups. This analysis resumed 100% of the variance in the two first axes based on the same parameters selected in the horse groups analysis (Fig 3).

### Genetic analysis

The genetic structure analysis based on microsatellites showed a genetic difference between CPF group and diagonally gaited groups (CTR, CTRG, and CTG groups) (S1 Fig). Also, the AMOVA showed a genetic difference of 5.3% (P = 0.02) between CPF group and diagonally gaited groups.

The frequency of the mutant *DMRT3* A-allele in the whole CPH breed was 0.3 but there was a significant difference in the genotype distribution among the 4 horse groups (P = 2.2 x10^-*16*^; Table 4). The mutant A-allele was fixed in the CPF group and the frequency of the mutation in the sample of the diagonally gaited horses (CTR, CTRG, and CTG group) was 0.04. The homozygous AA genotype was not found in the diagonally gaited horses. Also, there was a heterozygote excess (P<0.001) in the diagonally gaited horses (CTR, CTRG, and CTG group). In addition, the number of horses which performed diagonal gaits with different *DMRT3* genotypes that performed a clear or unclear gait footfall pattern is presented in Table 5.

**Table 4:** Genotype frequencies of the *DMRT3* mutation in a sample of Colombian paso horses.

Colombia paso horse group	*n*	AA	CA	CC	P
Colombian paso fino	42	1.00	0.00	0.00	
Colombian trocha	50	0.00	0.02	0.98	
Colombian trocha and gallop	27	0.00	0.07	0.93	
Colombian trot and gallop	33	0.00	0.15	0.85	
Total	152	0.28	0.05	0.67	2.2 x10^-*16*^

**Table 5:** Number of diagonally gaited horses with different *DMRT3* genotypes that perform a clear or unclear gait footfall pattern.

Diagonal gaits	CA		CC		P
	Clear gait	Unclear gait	Clear gait	Unclear gait	
Trocha	3	0	5	47	0.002
Colombian trot	3	0	14	7	0.529

## Discussion

### Asymmetry

The asymmetries identified in some of the kinematic parameters could be explained by a possible lameness of the limb where the fetlock extension, carpal flexion or tarsal flexion angle was increased. An angle increase in joint flexion or extension (Fig 1) is associated with less weight bearing, which may be a sign of lameness [31]. However, it is uncertain if the asymmetries identified within our study are associated with pain (lameness) or natural variation. In a recent study [32], 222 horses were analyzed in training and supposed to be sound by the owners, and still, 73% of them showed movement asymmetries. To evaluate lameness in our horses, additional measurements and analysis are required as suggested by previous studies [33–35]. In addition, the selection of the kinematic parameters, based on asymmetries (the variables not affected by asymmetry were chosen), contributes to the reliability of the data used for the statistical analyses performed in the current study.

### Kinematic parameters of the CPH breed

The gaits (paso fino, trocha, and Colombian trot) performed by the CPH are distinctive for this breed. For all the gaits, there is always at least one limb in stance phase and there is a high stride frequency and short stride length. The stride frequencies for the paso fino (2.60–2.85 strides per second) and trocha gait (2.70–2.96 strides per second) are higher than for similar walking gaits as tölt and foxtrot (2.23–2.36 strides per second) [3], and comparable to the stride frequency at gallop in racing breeds like the Thoroughbred (2.27–2.92 strides per second) [3,36,37]. On the other hand, the stride length of the CPH gaits is shorter (0.64–0.85 m) than in other walking gaits like tölt and foxtrot [3]. These characteristics make it difficult to distinguish the CPH gaits by just using just the human eye, and even more difficult to evaluate the footfall pattern and the kinematic parameters of the CPH gaits. Therefore, the objective measurements presented in the current study can be useful for establishing judging parameters and genetic improvement programs in the CPH breed (S1 Table).

### Kinematic differences between the CPH gaits

The gaits of the CPH breed were different based on the kinematics traits and genetic data (*DMRT3* genotypes and microsatellites) analyzed in the current study. By using these genetic and kinematic parameters, it was possible to clearly distinguish the gaits. Regarding the kinematic data, most of the gait differences could be explained by the stride frequency as well as the elbow and fetlock flexions, protraction and retraction measurements. Also, in the current study we have demonstrated that trocha gait can be considered the same gait for both CTR and CTRG horse groups. The only difference between CTR and CTRG horse groups was found for the stride frequency and the fetlock front speed (CTRG is slower), except for the fact that the CTRG horses are also trained to perform canter in competitions.

The Colombian trot gait is the most differentiated gait among the CPH gaits. It had the lowest stride frequency, flexion and extensions angles, and the highest protraction and retraction values. The paso fino and trocha gaits were more corresponding to each other than to the Colombian trot. The stride frequency of paso fino and trocha gait was similar and both gaits have a lateral sequence footfall pattern. However, the main difference between these two gaits is that paso fino is a lateral coupled gait whereas trocha is a diagonal coupled gait. Moreover, there were significant differences in joint flexions and extensions (paso fino is higher) between the paso fino and the trocha gait.

### Genetic differences in the CPH gaits

In addition to the kinematic differences, we observed a genetic differentiation of two groups based on the microsatellite data of the horses that also were genotyped for the *DMRT3* mutation: the CPF group and the diagonally gaited horses (CTR, CTRG and CTG groups) (S1 Fig), as previously reported in a study using the microsatellite census data [17]. This genetic structure also corresponded to two *DMRT3* groups: horses with the AA genotype of the *DMRT3* mutation (horses performing the paso fino gait) and horses with CC and CA genotypes (horses performing the trocha and the Colombian trot gaits). The genetic differences between the CPF group and the other CPH groups likely reflect the selection process for the last 50 years mainly based on the gaits present in the CPH.

Furthermore, there were differences in the frequency of the *DMRT3* mutant A-allele in the current study compared to a previous report [15] for the CPF (0.94 vs 1 in the current study), the CTR (0.10 vs 0.01 in the current study), and the CTG (0.14 vs 0.07 in the current study) groups (the CTRG group was not used for the comparison since there were only 4 horses in the previous study). The differences in the mutant A-allele frequencies between the two studies could possibly be explained by a different classification of the horse groups and possibly the relatedness among the horses differ between the two studies [15].

In the current study, the mutant A-allele of the *DMRT3* gene was significantly associated (P = 2.2 x10^-16^) with the horses’ ability to perform the paso fino gait, where it seems like the AA genotype is required for a horse to perform this gait. In our sample, the mutation was fixed in the CPF group and it was found in low frequencies in the other horse groups. The high frequency of the mutation in the CPF group could be explained by a high artificial selection pressure in the CPF horses, considering that the paso fino horses were separated from the trocha and trot horses in competitions already in the 1980´s (Fedequinas, personal communication), and the breeders have been selecting horses with a clear paso fino gait.

On the other hand, it seems to be a selection against the *DMRT3* AA genotype in horses performing the trocha and Colombian trot. The selection of the trocha gait appears favoring the CC genotype indicating that *DMRT3* is likely not the most important gene responsible for controlling the lateral footfall pattern in this gait. In addition, similar results have been found in the Mangalarga marchador horse breed in Brazil [6]. Although the CC genotype appears to be the most favorable for the trocha gait, there were a few horses with the CA genotype. The presence of the A-allele in horses performing the trocha and Colombian trot gaits could possibly be explained by the fact that all CHP horses share a common origin [2], and that there has not been enough time since the gait selection started, for the A-allele to completely disappear from the population of horses performing the trocha and trot gait. Also, there was a tendency where the CA genotype partly explains the horses’ ability to perform a clear footfall pattern in the trocha gait (P = 0.002; Table 5), however this hypothesis must be further explored.

The ambivalent position of the trocha gait is interesting. Based on the kinematic measurements, the trocha gait is closer to the paso fino gait than to the trot gait (Fig 6), and the trocha gait is not explained by the *DMRT3* mutant A-allele as it is for the paso fino gait (Table 4). Since the trocha gait is classified as a stepping gait, with a lateral sequence footfall pattern in diagonal couplets [7], two options are proposed to explain the nature of the trocha gait. The first hypothesis is that the trocha gait is an artificial gait (not inherited but conditioned by training) and could be considered as a dissociation from trot. The second hypothesis is that the trocha gait is a natural gait (inherited), and that there are other genes that explain this gait.

According to the first hypothesis, a previous study [38] showed that “at moderate speeds individual horses use dissociation patterns that allow them to maintain trunk pitch stability through management of the cranio-caudal location of the COP” (center of pressure), [38]. Those dissociations may have mechanical advantages over synchronous contacts in certain circumstances, and as trotting speed increases, forelimb vertical peak force increases, and dissociations tend towards hind-first [38], but no to fore-first dissociations, which is the case for the trocha gait. In addition, similar results have been found in Icelandic horses when the speed increases. In that circumstance, the tölt gait tends towards lateral couplets (hind-first), but tölt with diagonal couplets was rarely presented [39]. Therefore, it seems that a gait with diagonal couplets or fore-first pattern (as the trocha gait), is not completely explained as a dissociation from trot or a deviation from a lateral couplet gait as the tölt gait is in Icelandic horses.

Regarding the second hypothesis, additional data provided by Fedequinas was analyzed, consisting of 2919 horses whose parents and grandparents all performed the trocha gait, all of them parentage tested. The registers showed that 94.75% of those 2919 offspring had the ability to perform the trocha gait, supporting the idea that the trocha is an inherited gait. Furthermore, there are other gaits like the foxtrot (Missouri fox trotter) and the marcha batida (Mangalarga marchador) gaits which have been described with the same footfall pattern as the trocha gait. However, in contrast to horses performing the trocha gait, the *DMRT3* mutant A-allele is fixed in horses performing foxtrot (100% vs 1% in horses performing the trocha gait) [15]. On the other hand, the *DMRT3* C-allele is fixed in horses performing the marcha batida gait as well as in horses performing the trocha gait, and a recent study proposed that there are likely other genetic mechanisms that explain the marcha batida gait [40]. This also support the hypothesis that the trocha is an inherited gait, and that there are other genes than *DMRT3*, or other mutations in the *DMRT3* gene, that influences these diagonally stepping gaits in horses.

### Conclusions

The gaits within the CPH breed can be classified in different groups, using both kinematic data (stride frequency, fetlock extension and flexion, tarsal flexion, carpal flexion, fetlock front and hock speed measurements, and footfall pattern) and genetic data (microsatellite and *DMRT3* genotype frequencies). This makes it possible to implement genetic improvement programs and to establish kinematic parameters for each gait. Our data supports the hypothesis that the selection has produced kinematic differences between the Colombian paso horse’s gaits, particularly between the Colombian trot and the other gaits (the paso fino and trocha gait). Also, the *DMRT3* mutation seems to explain the horses’ ability to perform the paso fino gait but not the other diagonal coupled gaits (trocha and Colombian trot). However, there were no microsatellite or *DMRT3* genotype differences between horses performing the trocha and the Colombian trot gait. We propose that trocha is an inherited gait and its ambivalent position could be explained by other genes than the *DMRT3* gene, or other mutations in this gene, that influences this diagonally stepping gait. Therefore, it is very likely that other genetic factors are involved in regulating the trocha and the Colombian trot gaits in CPH horses. Finally, this study may serve as a foundation for implementing a genetic selection program in the Colombian paso horse and future gene discovery studies for locomotion pattern in horses.

## Supporting information

S1 Video Colombian paso fino horse performing paso fino gait. Reprinted under a CC BY license, with permission from Héctor Barriga Torres, original copyright [2018].
(MP4)

S2 Video Colombian trocha horse performing trocha gait. Reprinted under a CC BY license, with permission from Héctor Barriga Torres, original copyright [2018].
(MP4)

S3 Video Colombian trot and gallop horse performing Colombian trot gait. Reprinted under a CC BY license, with permission from Héctor Barriga Torres, original copyright [2018].
(MP4)

S4 Video Colombian trot and gallop horse performing gallop. Reprinted under a CC BY license, with permission from Héctor Barriga Torres, original copyright [2018].
(MP4)

S1 Table Mean and variation for kinematic parameters by horse group and sex in a sample of Colombian paso horse breed. (DOCX)

S1 Fig Genetic structure analysis based on microsatellites of the Colombian paso horse sample. Inferred ancestry of individuals (Y-axis) per horse (bar columns) in the CPH groups (X-axis). 1) Colombian trot and gallop group. 2) Colombian trocha and gallop group. 3) Colombian trocha group. 4) Colombian paso fino group.
(TIF)
