# Correction: Selection on the Colombian paso horse's gaits has produced kinematic differences partly explained by the *DMRT3* gene

**DOI:** 10.1371/journal.pone.0212149

**Published:** 2019-02-06

**Authors:** Miguel Novoa-Bravo, Kim Jäderkvist Fegraeus, Marie Rhodin, Eric Strand, Luis Fernando García, Gabriella Lindgren

The penultimate sentence of the second paragraph of the Introduction is incorrect. The correct sentence is: For each competition, the horses are separated by horse group (CPF, CTR, CTRG or CTG), sex and age (three categories: 36–48 months, 48–60 months, and more than 60 months), and evaluated by three judges.

S3 Video is incorrect. Please see the correct video here.

## Supporting information

S3 Video Colombian trot and gallop horse performing Colombian trot gait. Reprinted under a CC BY license, with permission from Héctor Barriga Torres, original copyright [2018].
(MP4)
