# Supplementary material for: Selection on the Colombian paso horse's gaits has produced kinematic differences partly explained by the DMRT3 gene
Source: PLoS One. 2018 Aug 17;13(8):e0202584. doi: 10.1371/journal.pone.0202584 (PMC6097835; doi:10.1371/journal.pone.0202584)
Supplement: S1 Table — (DOCX) [file pone.0202584.s005.docx]

S1 Table. Mean and variation for kinematic parameters by horse group and sex in a sample of Colombian paso horse breed.

| **Parameter** | **Group** | **Sex** | **N** | **Mean** | **CI 95%** | **SE** | **SD** | **CV (%)** | **Skwe.** | **Kurt.** |
| --- | --- | --- | --- | --- | --- | --- | --- | --- | --- | --- |
| *Fetlock flexion - front (P1-P2-P3)* | CPF | Female | 13 | 137.48 | (133.17-141.78) | 6.84 | 1.98 | 5.18 | 0.31 | -1.26 |
| *Fetlock flexion - front (P1-P2-P3)* | CPF | Male | 17 | 133.35 | (127.91-138.8) | 10.28 | 2.57 | 7.95 | -0.26 | -0.42 |
| *Fetlock flexion - front (P1-P2-P3)* | CTR | Female | 8 | 127.43 | (120.9-133.95) | 7.30 | 2.76 | 6.12 | -0.06 | -1.41 |
| *Fetlock flexion - front (P1-P2-P3)* | CTR | Male | 12 | 125.82 | (120.9-130.74) | 7.41 | 2.24 | 6.15 | 0.13 | -1.47 |
| *Fetlock flexion - front (P1-P2-P3)* | CTRG | Female | 6 | 130.47 | (118.12-142.82) | 10.74 | 4.80 | 9.02 | 0.37 | -1.04 |
| *Fetlock flexion - front (P1-P2-P3)* | CTRG | Male | 10 | 126.32 | (119.37-133.27) | 9.21 | 3.07 | 7.69 | 0.19 | -1.63 |
| *Fetlock flexion - front (P1-P2-P3)* | CTG | Female | 9 | 125.76 | (119.77-131.74) | 7.34 | 2.60 | 6.19 | -0.64 | -1.44 |
| *Fetlock flexion - front (P1-P2-P3)* | CTG | Male | 9 | 127.71 | (121.39-134.03) | 7.75 | 2.74 | 6.44 | -0.08 | -1.45 |
| *Fetlock extension - front (P1-P2-P3)* | CPF | Female | 17 | 123.40 | (119.99-126.81) | 6.43 | 1.61 | 5.37 | -0.36 | -1.42 |
| *Fetlock extension - front (P1-P2-P3)* | CPF | Male | 22 | 124.07 | (121.44-126.71) | 5.80 | 1.27 | 4.79 | 0.58 | -0.44 |
| *Fetlock extension - front (P1-P2-P3)* | CTR | Female | 20 | 118.40 | (115.23-121.57) | 6.61 | 1.52 | 5.73 | -0.20 | -0.63 |
| *Fetlock extension - front (P1-P2-P3)* | CTR | Male | 22 | 122.08 | (119.29-124.87) | 6.15 | 1.34 | 5.16 | -0.05 | -1.11 |
| *Fetlock extension - front (P1-P2-P3)* | CTRG | Female | 15 | 118.60 | (115.17-122.03) | 5.99 | 1.60 | 5.23 | 0.24 | -0.23 |
| *Fetlock extension - front (P1-P2-P3)* | CTRG | Male | 15 | 122.35 | (119.35-125.35) | 5.23 | 1.40 | 4.43 | 0.19 | 0.14 |
| *Fetlock extension - front (P1-P2-P3)* | CTG | Female | 13 | 117.52 | (113.55-121.49) | 6.31 | 1.82 | 5.59 | -0.02 | -1.13 |
| *Fetlock extension - front (P1-P2-P3)* | CTG | Male | 20 | 121.20 | (117.89-124.51) | 6.89 | 1.58 | 5.83 | -1.38 | 2.73 |
| *Carpal flexion (P2-P3-P4)* | CPF | Female | 16 | 119.43 | (115.71-123.14) | 6.75 | 1.74 | 5.83 | -0.50 | -0.78 |
| *Carpal flexion (P2-P3-P4)* | CPF | Male | 24 | 122.43 | (120.52-124.35) | 4.43 | 0.92 | 3.70 | -0.17 | -1.04 |
| *Carpal flexion (P2-P3-P4)* | CTR | Female | 21 | 109.55 | (105.34-113.77) | 9.04 | 2.02 | 8.46 | 0.01 | -0.75 |
| *Carpal flexion (P2-P3-P4)* | CTR | Male | 22 | 115.34 | (112.53-118.15) | 6.19 | 1.35 | 5.50 | -0.11 | 0.30 |
| *Carpal flexion (P2-P3-P4)* | CTRG | Female | 15 | 107.73 | (102.58-112.89) | 8.99 | 2.40 | 8.64 | 0.31 | -0.68 |
| *Carpal flexion (P2-P3-P4)* | CTRG | Male | 15 | 112.29 | (108.96-115.63) | 5.82 | 1.56 | 5.37 | -0.64 | 0.05 |
| *Carpal flexion (P2-P3-P4)* | CTG | Female | 15 | 109.73 | (104.02-115.44) | 9.96 | 2.66 | 9.40 | 0.23 | -1.50 |
| *Carpal flexion (P2-P3-P4)* | CTG | Male | 20 | 112.94 | (110.02-115.86) | 6.08 | 1.39 | 5.52 | -0.14 | -0.12 |
| *Elbow flexion (P3-P4-P5)* | CPF | Female | 16 | 90.25 | (87.21-93.29) | 5.53 | 1.43 | 6.33 | -0.07 | -1.16 |
| *Elbow flexion (P3-P4-P5)* | CPF | Male | 20 | 88.84 | (86.39-91.29) | 5.10 | 1.17 | 5.89 | -0.30 | -0.62 |
| *Elbow flexion (P3-P4-P5)* | CTR | Female | 19 | 81.39 | (78.79-83.99) | 5.25 | 1.24 | 6.62 | 0.18 | -1.61 |
| *Elbow flexion (P3-P4-P5)* | CTR | Male | 17 | 83.19 | (80.24-86.14) | 5.57 | 1.39 | 6.90 | 1.13 | 0.50 |
| *Elbow flexion (P3-P4-P5)* | CTRG | Female | 14 | 80.81 | (77.75-83.88) | 5.12 | 1.42 | 6.58 | 0.23 | -1.59 |
| *Elbow flexion (P3-P4-P5)* | CTRG | Male | 13 | 82.32 | (77.8-86.84) | 7.18 | 2.07 | 9.08 | -0.13 | -1.55 |
| *Elbow flexion (P3-P4-P5)* | CTG | Female | 13 | 78.42 | (73.18-83.65) | 8.32 | 2.40 | 11.05 | 0.70 | -0.79 |
| *Elbow flexion (P3-P4-P5)* | CTG | Male | 18 | 78.47 | (74.37-82.56) | 8.01 | 1.94 | 10.50 | 0.45 | 0.53 |
| *Fetlock flexion - hind (P7-P8-P9)* | CPF | Female | 12 | 107.05 | (102.3-111.8) | 7.15 | 2.16 | 6.98 | 0.18 | -1.26 |
| *Fetlock flexion - hind (P7-P8-P9)* | CPF | Male | 14 | 109.07 | (103.37-114.77) | 9.51 | 2.64 | 9.05 | 0.03 | -1.13 |
| *Fetlock flexion - hind (P7-P8-P9)* | CTR | Female | 8 | 101.88 | (96.19-107.56) | 6.36 | 2.40 | 6.68 | -0.04 | -1.54 |
| *Fetlock flexion - hind (P7-P8-P9)* | CTR | Male | 12 | 104.65 | (101.78-107.52) | 4.33 | 1.30 | 4.32 | 0.02 | -1.32 |
| *Fetlock flexion - hind (P7-P8-P9)* | CTRG | Female | 6 | 104.87 | (101-108.74) | 3.37 | 1.51 | 3.52 | 0.63 | -1.04 |
| *Fetlock flexion - hind (P7-P8-P9)* | CTRG | Male | 10 | 103.70 | (97.1-110.3) | 8.75 | 2.92 | 8.89 | 0.02 | -1.38 |
| *Fetlock flexion - hind (P7-P8-P9)* | CTG | Female | 9 | 94.36 | (87.02-101.69) | 9.00 | 3.18 | 10.12 | 1.11 | 0.48 |
| *Fetlock flexion - hind (P7-P8-P9)* | CTG | Male | 10 | 101.40 | (92.68-110.12) | 11.57 | 3.86 | 12.03 | -0.02 | -1.07 |
| *Fetlock extension - hind (P7-P8-P9)* | CPF | Female | 14 | 123.69 | (119.99-127.4) | 6.19 | 1.72 | 5.19 | 0.23 | -0.68 |
| *Fetlock extension - hind (P7-P8-P9)* | CPF | Male | 24 | 123.86 | (120.7-127.01) | 7.31 | 1.52 | 6.03 | -1.29 | 1.38 |
| *Fetlock extension - hind (P7-P8-P9)* | CTR | Female | 22 | 119.13 | (116.58-121.68) | 5.62 | 1.23 | 4.83 | 0.02 | -1.46 |
| *Fetlock extension - hind (P7-P8-P9)* | CTR | Male | 22 | 118.60 | (115.74-121.45) | 6.29 | 1.37 | 5.43 | 0.20 | -0.80 |
| *Fetlock extension - hind (P7-P8-P9)* | CTRG | Female | 15 | 117.12 | (112.55-121.69) | 7.97 | 2.13 | 7.04 | -0.12 | -0.95 |
| *Fetlock extension - hind (P7-P8-P9)* | CTRG | Male | 13 | 117.63 | (114.06-121.2) | 5.68 | 1.64 | 5.03 | -0.49 | -0.84 |
| *Fetlock extension - hind (P7-P8-P9)* | CTG | Female | 17 | 116.53 | (112.94-120.12) | 6.78 | 1.69 | 6.00 | 0.28 | -1.31 |
| *Fetlock extension - hind (P7-P8-P9)* | CTG | Male | 20 | 118.07 | (114.87-121.27) | 6.65 | 1.53 | 5.78 | -0.15 | -0.45 |
| *Tarsal flexion (P8-P9-P10)* | CPF | Male | 21 | 92.99 | (90.21-95.77) | 5.97 | 1.33 | 6.58 | 0.21 | -0.70 |
| *Tarsal flexion (P8-P9-P10)* | CTR | Female | 22 | 86.78 | (82.89-90.68) | 8.58 | 1.87 | 10.12 | 0.14 | -0.62 |
| *Tarsal flexion (P8-P9-P10)* | CTR | Male | 20 | 90.77 | (87.44-94.1) | 6.93 | 1.59 | 7.83 | -0.03 | -0.89 |
| *Tarsal flexion (P8-P9-P10)* | CTRG | Female | 15 | 87.59 | (83.79-91.38) | 6.62 | 1.77 | 7.82 | -0.85 | -0.15 |
| *Tarsal flexion (P8-P9-P10)* | CTRG | Male | 13 | 88.60 | (84.89-92.31) | 5.91 | 1.71 | 6.94 | 0.32 | -1.12 |
| *Tarsal flexion (P8-P9-P10)* | CTG | Female | 15 | 81.80 | (79.08-84.52) | 4.75 | 1.27 | 6.01 | -0.05 | -1.00 |
| *Tarsal flexion (P8-P9-P10)* | CTG | Male | 19 | 86.11 | (82.19-90.02) | 7.90 | 1.86 | 9.43 | -0.43 | -0.37 |
| *Stride frequency* | CPF | Female | 17 | 165.66 | (160.28-171.04) | 10.16 | 2.54 | 6.32 | 0.45 | 0.35 |
| *Stride frequency* | CPF | Male | 25 | 160.86 | (156.49-165.23) | 10.38 | 2.12 | 6.58 | 0.02 | -0.42 |
| *Stride frequency* | CTR | Female | 22 | 165.94 | (162.42-169.45) | 7.74 | 1.69 | 4.77 | 0.29 | -0.49 |
| *Stride frequency* | CTR | Male | 22 | 172.76 | (167.33-178.18) | 11.95 | 2.61 | 7.08 | -0.06 | -0.86 |
| *Stride frequency* | CTRG | Female | 15 | 159.72 | (156.08-163.35) | 6.34 | 1.69 | 4.11 | -0.14 | -1.54 |
| *Stride frequency* | CTRG | Male | 10 | 163.31 | (157.6-169.03) | 7.58 | 2.53 | 4.89 | -0.37 | -1.49 |
| *Stride frequency* | CTG | Female | 17 | 130.05 | (125.18-134.92) | 9.19 | 2.30 | 7.28 | 0.19 | -1.36 |
| *Stride frequency* | CTG | Male | 20 | 137.07 | (133.28-140.86) | 7.90 | 1.81 | 5.91 | 0.02 | -0.20 |
| *Fetlock front speed* | CPF | Female | 15 | 32.31 | (29.97-34.64) | 4.08 | 1.09 | 13.07 | 0.06 | -0.90 |
| *Fetlock front speed* | CPF | Male | 22 | 32.05 | (29.85-34.24) | 4.83 | 1.05 | 15.44 | -0.43 | -0.74 |
| *Fetlock front speed* | CTR | Female | 22 | 39.71 | (36.14-43.28) | 7.87 | 1.72 | 20.28 | 0.45 | -0.83 |
| *Fetlock front speed* | CTR | Male | 22 | 37.09 | (33.34-40.84) | 8.27 | 1.80 | 22.82 | 0.55 | -0.90 |
| *Fetlock front speed* | CTRG | Female | 15 | 40.76 | (35.72-45.8) | 8.79 | 2.35 | 22.33 | 0.05 | -1.18 |
| *Fetlock front speed* | CTRG | Male | 12 | 44.30 | (39.09-49.51) | 7.84 | 2.37 | 18.50 | -0.03 | -0.23 |
| *Fetlock front speed* | CTG | Female | 14 | 36.83 | (33.1-40.56) | 6.22 | 1.73 | 17.53 | 0.61 | -0.32 |
| *Fetlock front speed* | CTG | Male | 19 | 34.24 | (31.13-37.35) | 6.28 | 1.48 | 18.84 | 0.42 | -1.25 |
| *Fetlock hind speed* | CPF | Female | 16 | 27.33 | (24.7-29.95) | 4.78 | 1.23 | 18.05 | 0.94 | 0.92 |
| *Fetlock hind speed* | CPF | Male | 23 | 27.72 | (25.35-30.09) | 5.36 | 1.14 | 19.78 | 0.16 | -0.78 |
| *Fetlock hind speed* | CTR | Female | 21 | 31.31 | (28.48-34.15) | 6.09 | 1.36 | 19.91 | -0.02 | -0.96 |
| *Fetlock hind speed* | CTR | Male | 20 | 29.03 | (26.49-31.57) | 5.28 | 1.21 | 18.66 | 0.21 | -0.82 |
| *Fetlock hind speed* | CTRG | Female | 13 | 30.46 | (26.75-34.17) | 5.89 | 1.70 | 20.14 | 0.20 | -1.30 |
| *Fetlock hind speed* | CTRG | Male | 14 | 33.87 | (30.13-37.62) | 6.25 | 1.73 | 19.15 | -0.40 | -1.28 |
| *Fetlock hind speed* | CTG | Female | 12 | 29.98 | (26.62-33.34) | 5.06 | 1.53 | 17.63 | 0.52 | -1.18 |
| *Fetlock hind speed* | CTG | Male | 19 | 29.07 | (26.96-31.19) | 4.27 | 1.01 | 15.08 | -0.12 | -0.82 |
| *Hock speed* | CPF | Female | 17 | 15.64 | (13.94-17.33) | 3.20 | 0.80 | 21.10 | 0.10 | -0.75 |
| *Hock speed* | CPF | Male | 23 | 14.51 | (13.32-15.7) | 2.69 | 0.57 | 18.94 | 0.26 | -0.60 |
| *Hock speed* | CTR | Female | 20 | 18.04 | (16.8-19.28) | 2.58 | 0.59 | 14.68 | 0.08 | -0.34 |
| *Hock speed* | CTR | Male | 21 | 17.95 | (16.06-19.84) | 4.05 | 0.91 | 23.11 | 0.16 | -0.87 |
| *Hock speed* | CTRG | Female | 14 | 17.86 | (15.7-20.01) | 3.60 | 1.00 | 20.90 | 0.97 | 0.03 |
| *Hock speed* | CTRG | Male | 15 | 19.44 | (17.82-21.06) | 2.83 | 0.76 | 15.07 | -0.11 | -0.79 |
| *Hock speed* | CTG | Female | 14 | 16.70 | (15.02-18.38) | 2.81 | 0.78 | 17.46 | 0.59 | -0.98 |
| *Hock speed* | CTG | Male | 19 | 15.63 | (14.44-16.82) | 2.40 | 0.57 | 15.79 | 0.51 | -0.82 |
| *Protraction* | CPF | Female | 11 | 20.82 | (19.95-21.68) | 1.23 | 0.39 | 6.18 | 0.05 | -1.39 |
| *Protraction* | CPF | Male | 17 | 21.41 | (20.7-22.11) | 1.32 | 0.33 | 6.37 | 0.06 | -0.51 |
| *Protraction* | CTR | Female | 13 | 23.10 | (21.8-24.4) | 2.07 | 0.60 | 9.31 | -0.43 | -0.88 |
| *Protraction* | CTR | Male | 14 | 22.55 | (21.32-23.78) | 2.05 | 0.57 | 9.45 | 0.62 | -0.61 |
| *Protraction* | CTRG | Female | 13 | 21.36 | (20.48-22.24) | 1.40 | 0.40 | 6.83 | -0.12 | -0.37 |
| *Protraction* | CTRG | Male | 12 | 22.00 | (20.82-23.18) | 1.78 | 0.54 | 8.44 | 0.24 | -1.43 |
| *Protraction* | CTG | Female | 9 | 22.29 | (20.99-23.59) | 1.59 | 0.56 | 7.57 | 0.46 | -1.37 |
| *Protraction* | CTG | Male | 15 | 22.95 | (21.94-23.95) | 1.75 | 0.47 | 7.89 | -0.32 | -1.06 |
| *Retraction* | CPF | Female | 11 | 17.35 | (15.75-18.96) | 2.28 | 0.72 | 13.77 | -0.26 | -1.52 |
| *Retraction* | CPF | Male | 17 | 16.56 | (15.46-17.66) | 2.08 | 0.52 | 12.92 | 0.64 | -0.64 |
| *Retraction* | CTR | Female | 13 | 18.11 | (16.49-19.73) | 2.58 | 0.74 | 14.82 | 0.30 | -1.24 |
| *Retraction* | CTR | Male | 14 | 16.91 | (15.38-18.45) | 2.56 | 0.71 | 15.71 | -0.51 | -0.55 |
| *Retraction* | CTRG | Female | 13 | 16.69 | (15.19-18.19) | 2.39 | 0.69 | 14.89 | -0.60 | -1.30 |
| *Retraction* | CTRG | Male | 12 | 16.53 | (13.98-19.09) | 3.85 | 1.16 | 24.35 | -0.48 | -0.61 |
| *Retraction* | CTG | Female | 9 | 18.51 | (17.03-19.99) | 1.82 | 0.64 | 10.42 | -0.11 | -1.51 |
| *Retraction* | CTG | Male | 15 | 18.62 | (17.36-19.88) | 2.19 | 0.59 | 12.18 | -0.07 | -0.93 |
| *Stride length front* | CPF | Female | 17 | 68.42 | (56.48-80.37) | 22.54 | 5.63 | 33.95 | -0.27 | -1.10 |
| *Stride length front* | CPF | Male | 24 | 63.50 | (56.4-70.6) | 16.46 | 3.43 | 26.48 | 0.20 | -1.16 |
| *Stride length front* | CTR | Female | 22 | 85.29 | (75.35-95.23) | 21.91 | 4.78 | 26.29 | 0.22 | -1.18 |
| *Stride length front* | CTR | Male | 21 | 70.62 | (60.5-80.74) | 21.69 | 4.85 | 31.47 | 0.30 | -1.38 |
| *Stride length front* | CTRG | Female | 11 | 85.08 | (72.27-97.89) | 18.17 | 5.74 | 22.40 | -0.17 | -1.02 |
| *Stride length front* | CTRG | Male | 15 | 71.93 | (62.95-80.92) | 15.67 | 4.19 | 22.56 | 0.16 | -1.64 |
| *Stride length front* | CTG | Female | 17 | 81.75 | (73.12-90.38) | 16.28 | 4.07 | 20.53 | -0.19 | -0.91 |
| *Stride length front* | CTG | Male | 20 | 62.19 | (55.84-68.53) | 13.21 | 3.03 | 21.80 | 0.01 | -0.31 |
| *Stride length hind* | CPF | Female | 17 | 69.48 | (57.57-81.4) | 22.48 | 5.62 | 33.35 | -0.40 | -1.24 |
| *Stride length hind* | CPF | Male | 24 | 64.74 | (57.85-71.62) | 15.96 | 3.33 | 25.18 | 0.03 | -1.35 |
| *Stride length hind* | CTR | Female | 22 | 86.44 | (76.9-95.97) | 21.00 | 4.58 | 24.87 | 0.21 | -1.19 |
| *Stride length hind* | CTR | Male | 21 | 71.31 | (61.24-81.38) | 21.58 | 4.83 | 31.01 | 0.37 | -1.45 |
| *Stride length hind* | CTRG | Female | 15 | 79.43 | (66.39-92.47) | 22.74 | 6.07 | 29.64 | -0.12 | -1.02 |
| *Stride length hind* | CTRG | Male | 15 | 73.52 | (64.24-82.8) | 16.18 | 4.32 | 22.78 | 0.19 | -1.62 |
| *Stride length hind* | CTG | Female | 17 | 82.71 | (74.78-90.65) | 14.97 | 3.74 | 18.66 | -0.34 | -1.27 |
| *Stride length hind* | CTG | Male | 20 | 63.64 | (56.74-70.53) | 14.35 | 3.29 | 23.14 | 0.17 | -0.80 |

Flexions, extensions, protraction, and retraction in degrees; stride frequency in strides per minute; speed in cm/s; stride lengths in cm. CPF-Colombian Paso Fino, CTR-Colombian Trocha, CTRG-Colombian Trocha and Gallop, CTG-Colombian Trot and Gallop. N, number of horses; CI, confidence interval; SE, standard error; SD, standard deviation; VC, variation coefficient; Skew., skewness; Kurt., kurtosis; The positions of the landmarks (P1-P10) are described in the Fig 1.
