# Supplementary figures and images for: Selection on the Colombian paso horse's gaits has produced kinematic differences partly explained by the DMRT3 gene
Source: PLoS One. 2018 Aug 17;13(8):e0202584. doi: 10.1371/journal.pone.0202584 (PMC6097835; doi:10.1371/journal.pone.0202584)

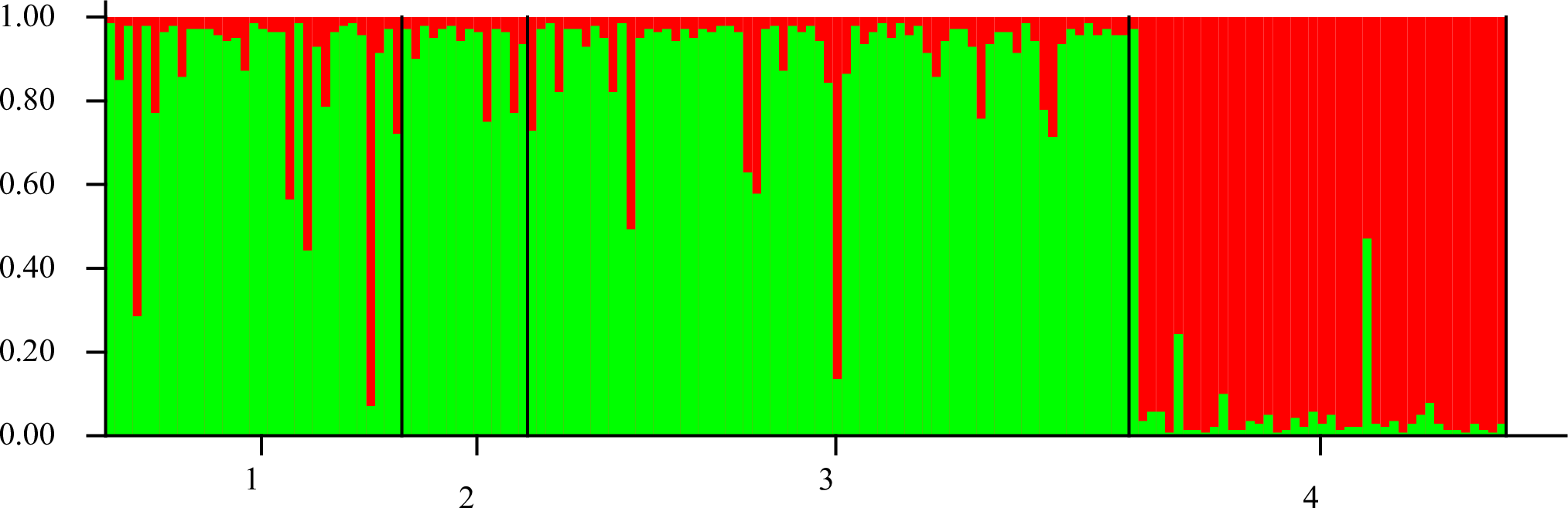

Supplement: S1 Fig — Inferred ancestry of individuals (Y-axis) per horse (bar columns) in the CPH groups (X-axis). 1) Colombian trot and gallop group. 2) Colombian trocha and gallop group. 3) Colombian trocha group. 4) Colombian paso fino group. (TIF) [file pone.0202584.s006.tif]
